# Prebiotically Plausible Vesicle Populations Can Respond to Selection for Greater Turbidity via Emergent Cooperative Dynamics

**DOI:** 10.1021/acs.langmuir.6c00275

**Published:** 2026-06-13

**Authors:** Tymofii Sokolskyi, David Baum

**Affiliations:** † Wisconsin Institute for Discovery, 5228 University of Wisconsin-Madison, Madison, Wisconsin 53715, United States; ‡ Department of Botany, University of Wisconsin-Madison, Madison, Wisconsin 53715, United States

## Abstract

Adaptive evolution has long been hypothesized to be possible in the absence of genetic molecules, but experimental evidence remains lacking. Fatty acid vesicles can spontaneously grow and divide and might therefore be capable of nongenetic inheritance, making them ideal for exploring the emergence of prebiotic evolution. In this study, we tested whether vesicle populations can respond to artificial selection for greater turbidity and, if so, whether that response can be tied to an inheritance-like mechanism. We prepared 192 independent vesicle populations, incubated them for 24 h, and then selected half of the populations to propagate into the next generation. The populations to propagate were picked either randomly, representing drift controls, or were those with the greatest turbidity, representing selection. Population propagation involved resuspension, transfer into fresh buffer, feeding with an amphiphile stock, and then incubating for the next 24 h. In three replicate experiments run for at least 10 generations, we observed consistently greater turbidity in selection compared with drift lineages. This was accompanied by a reduction in the heritability (the regression slope between parent and offspring turbidities) of the selected lineages. We conducted additional experiments to evaluate whether this response to selection is caused by a simple carryover effect or reflects cooperative dynamics where vesicles from a parental population affect newly formed vesicles in the next generation. The response to selection is much lower when the resuspension step was omitted and/or if new amphiphiles were provided as preformed vesicles. Combined with fluorescence analyses of the resuspension and feeding processes, these results suggest that cooperative vesicle dynamics occur in cases where a small number of intact vesicles from a parental generation interact with an excess of incorporated amphiphiles. Overall, this study represents the first experimental finding of a response to artificial selection in prebiotic chemistry.

## Graphical abstract

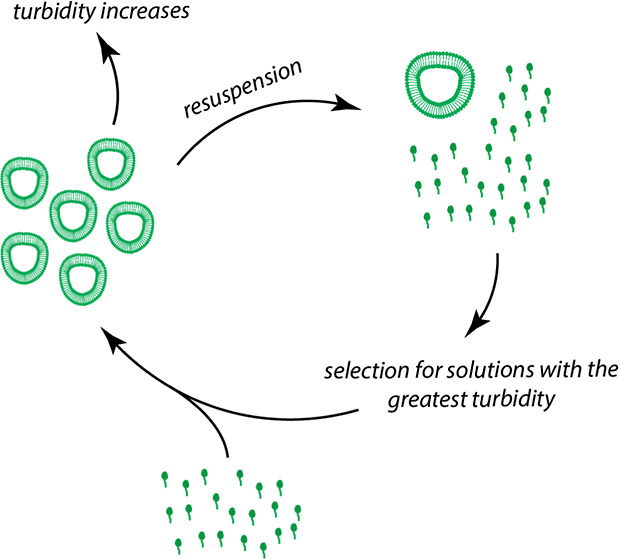

## Introduction

If one defines life as a self-sustaining chemical system capable of Darwinian evolution,^1^ explaining the origins of life requires that we find chemical systems that are capable of adaptive evolution yet simple enough to emerge spontaneously under prebiotic conditions.^2^ One class of models argues that the first evolvers were self-organized amphiphile assemblies (micelles, droplets, or vesicles) that could grow and divide and pass on their properties via compositional rather than genetic information encoding.^3–7^ Amphiphiles such as long-chain fatty acids are present on meteorites^8^ and comets,^9^ which likely delivered small amounts of amphiphiles to prebiotic oceans,^8^ and would have supplemented the spontaneous formation of similar molecules on Earth via hydrothermal processes.^10,11^ Thus, it is possible that amphiphile assemblies formed in some geological settings, such as in tectonic fault zones.^12^ Since fatty acid vesicles can grow and divide,^13,14^ they would be plausible candidates for the first adaptive evolvers provided they could generate heritable variation on which selection could act.

Spontaneously formed prebiotic vesicles could potentially manifest heritability via two distinct mechanisms. The first is through compositional inheritance based on the relative abundances of different vesicle-associated and/or encapsulated molecules, as suggested by the graded autocatalysis replication domain (GARD) model. The GARD model suggests that amphiphile assemblies could replicate and pass on compositional information to the following generations due to mutual catalysis, where assemblies are composed of amphiphile species that promote one another’s assembly.^3–7^ The second mechanism of inheritance is through a physical phenomenon called the matrix effect, where a set of pre-existing vesicles provided with an excess of unincorporated food cause the formation of new vesicles that are similar in size to the pre-existing vesicles.^15^ This phenomenon has been observed with varying food to template ratios and with mixtures of more than one amphiphile species.^16^ A proposed mechanism for the matrix effect is that the new amphiphiles insert rapidly into the outer membrane leaflet of a template vesicle, which causes curvature and eventually formation of a second vesicle of similar size to the template.^17^ The two mechanisms may not be completely independent because physical features of vesicles, such as size, shape, or lamellarity, can be influenced by diverse chemical species that might associated with vesicle membranes.^18–21^

Whether inheritance is based on composition or the matrix effect, we might expect that populations of vesicles subjected to selection for an emergent trait would change over time in the selected direction. The only previous experimental study in this area found weak and inconsistent evidence for heritability of size and Nile Red fluorescence in serially transferred vesicles composed of decanoic acid and decylamine.^22^ Additionally, some recent work points to the presence of ecology-like interactions among vesicle populations.^23^

Here, we tested whether populations of vesicles can respond to selection for an emergent trait, turbidity, as measured by light scattering at 400 nm. Turbidity is a multifactorial trait that is sensitive to several vesicle characteristics (radius, shape, lamellarity, and lumen contents).^24^ The diversity of traits influencing turbidity makes it an attractive target for selection because only one of those underlying traits needs to manifest heritable variation for a response to selection to be seen. It is also quick and easy to measure in a plate reader, which allows for large sample sizes. We found a significantly higher turbidity in selected versus unselected (drift) vesicle populations. This response to selection was associated with a loss of heritability, which may explain why the divergence between selected and unselected lines was limited to the first few generations. Follow-on experiments suggest that this response to selection cannot entirely be explained by carrying forward bulk composition. Instead, a matrix effect-like process provides the most plausible mechanism for the inheritance of vesicle characteristics from one generation to the next. Further work is needed, however, to elucidate the mechanism and see if a more consistent and open-ended response to selection would be seen if a greater diversity of amphiphiles was present.

## Experimental Section

### Vesicle Preparation

For vesicle stock preparation, we used decanoic acid or DA (Pfaltz & Bauer, Waterbury, CT, USA), 1-decanol or DOH (Santa Cruz Biotechnology, Dallas, TX, USA), and methanol (Fischer Scientific, Hampton, NH, USA). For the preparation of the 0.1 M pH 7 phosphate buffer (PB), we used monobasic sodium phosphate monohydrate (Millipore Sigma, Burlington, MA, USA) and anhydrous dibasic sodium phosphate (Dot Scientific, Burton, MI, USA). All pipetting of solutions containing vesicles was done with a p1000 pipet since unpublished preliminary experiments showed that smaller pipet tips disrupted vesicle integrity. Since different pipetting styles can affect the turbidity of vesicle populations, all transfers were done by the same person, the lead author.

### Experimental Setup

To test whether vesicle populations can respond to selection for greater turbidity, we compared changes in turbidity over generations with and without selection. Each experiment was conducted in two 96-well plates (Dot Scientific, Burton, MI, USA), one composed of selected lineages and the other of unselected (drift) lineages. In selected lineages, the 48 wells with highest turbidity were allowed to replicate (each was used to seed two wells in the next generation), while the 48 lowest scoring wells each left no descendants. In the drift controls, 48 randomly selected wells were replicated (Figure 1).

**Figure 1:**
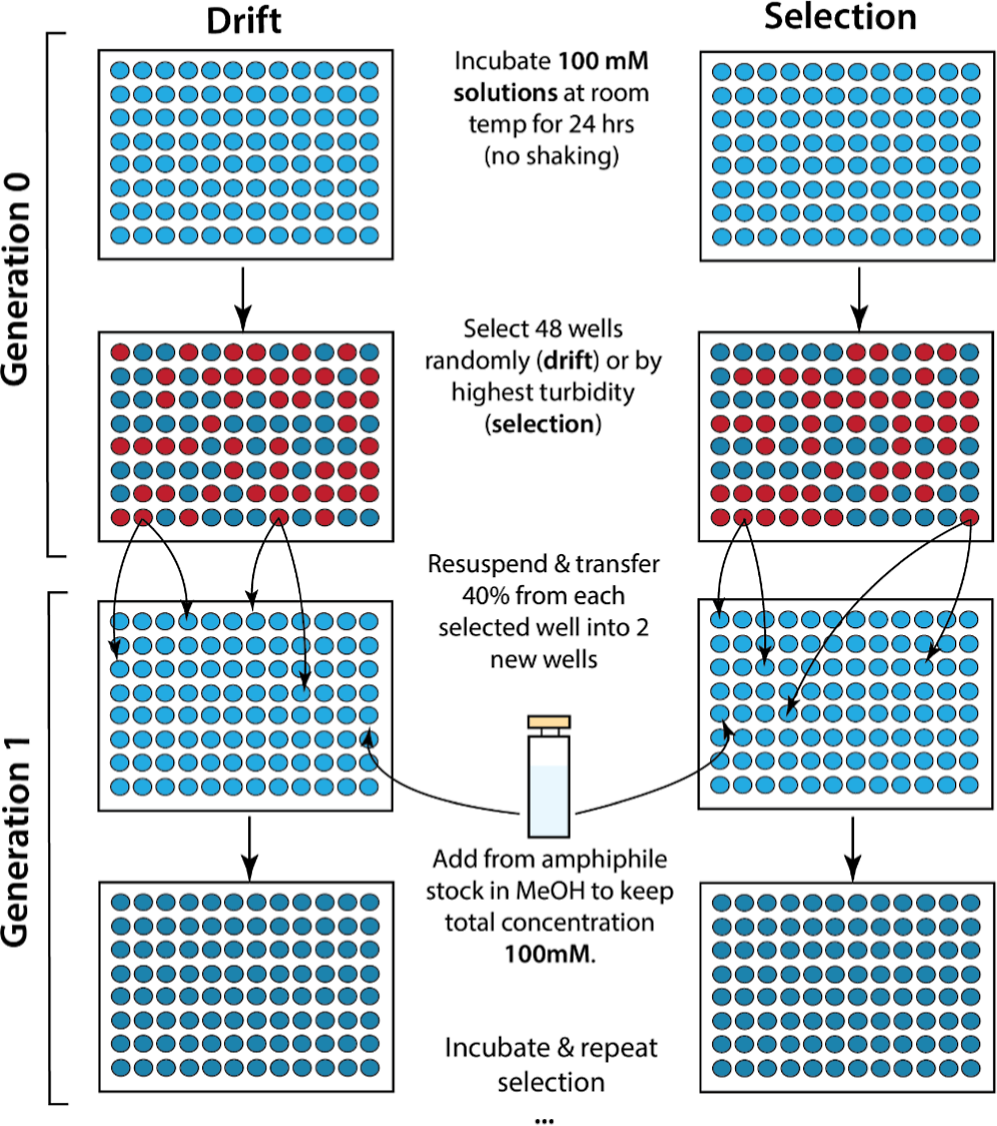
Scheme of selection and drift in the feeding-after-resuspension (FR) protocol.

The total volume in each well was always 200 μL. For all experiments, generation 0 (the generation before the first transfer) was initiated by the addition of 180 μL of PB to each well, followed by 20 μL of a 1 M 2:1 DA:DOH stock solution (in methanol). Then, each well was resuspended with a p1000 pipet 5 times and plates were sealed with a plastic seal (Thermo Fischer Scientific, Waltham, MA, USA), taped around the edges to prevent evaporation, and left on a bench at room temperature for 24 h. In all subsequent generations, 48 of the 96 wells on a plate were transferred into two wells of a new plate. For both drift and selection plates, “receiver” wells were randomized to control for position effects. At the end of each generation, just prior to transfers, turbidity was measured by a Biotek Synergy HTX (Agilent Technologies, Santa Clara, CA, USA) spectrophotometer as absorbance at 400 nm.

The same selection and drift experiments were conducted using four alternative protocols, which we call: fed and resuspended (FR), fed and unresuspended (FU), unfed and resuspended (UR), and unfed and unresuspended (UU). In FR experiments, food, in the form of amphiphile monomers in methanol, was added to vesicles from the previous generation (Figure 1). Each well of a plate from a prior generation was resuspended 5 times with a p1000 pipet and then 80 μL was transferred into 108 μL of PB in a receiver well on a new plate. Then, 12 μL of the 1 M 2:1 DA:DOH stock in methanol was added to each well on the new plate. We conducted 3 experiments of this type with an almost identical setup—FR1-3. FR1 lasted for 20 generations, while FR2 and FR3 ended after 10 generations. For FR3, we included 0.2 mM of Nile Red (Neta Scientific, Marlton, NJ, USA) in the amphiphile stock. Three replicate FU experiments were conducted, each for 10 generations, in a similar manner to FR except that the transfer population was not resuspended but was instead transferred with gentle pipetting using a p1000 pipet tip.

For UR transfers, resuspended transfer material from a previous generation was added to wells that contained preformed vesicles. To prepare the food vesicle solutions, a day before each transfer, 108 μL of PB was mixed with 12 μL of the 1 M amphiphile stock in each well of the receiver plate and resuspended 5 times. The plate was then sealed and left for 24 h to form vesicles. Each transferred well from the previous generation was resuspended five times and 80 μL was transferred into the prepared receiver plate. We conducted three experiments of this type, UR1-3, each for 10 generations.

For UU experiments, we prepared a vesicle food solution for each well a day before the transfer, as described for UR. However, in UU experiments, transfers involved gentle pipetting to keep vesicles as intact as possible. We conducted three replicate experiments of this type, UU1-3, each for 10 generations. UU1 and UU2 were done with the same generation 0 setup as FR1-3 and UR1-3, however, for UU3 we started with a generation 0 that received one 40% transfer and thus had similar turbidity to the values of FR1-3. We also conducted one 5 generation transfer experiment, UU4, which omitted the usual 24 h incubation; instead, transfers were conducted sequentially using food vesicles prepared the day before.

For each generation of each experiment, we calculated heritability (*h* ^2^) as it is defined in population genetics:^25^ the slope of the linear regression between parent and offspring traits. We unscrambled the randomization to determine specific parent-offspring pairs of wells for each generation and then calculated the slope with parent turbidities treated as independent variables. Greater positive slope values indicate greater heritability.

### Calcein Fluorescence

To determine the degree of vesicle leakage during the course of a 24 incubation and during pipetting between generations, we used calcein as a fluorescent probe. A 5 mM stock solution of calcein was prepared by dissolving the powder (Santa Cruz Biotechnology, Dallas, TX, USA) in methanol. This solution was added to PB to a final calcein concentration of 0.125 mM. The resulting solution was dispensed into 20 wells of a black 96-well plate and a 2:1 DA:DOH stock in methanol was added to a final concentration of 100 mM. Emission at 528 nm following excitation at 485 nm was measured every 30 min using the Biotek Synergy HTX (Agilent Technologies, Santa Clara, CA, USA) spectrophotometer. To prepare the positive control—a solution without vesicles but the same amphiphile concentrations—we added 1% of Triton X-100 (Santa Cruz Biotechnology, Dallas, TX, USA) to the calcein buffer and resuspended the wells after amphiphiles were added. This concentration of Triton is sufficient to prevent vesicle formation in these conditions.^22^ After 24 h, 40% of each of the sample was transferred into fresh PB (lacking calcein) in a new black plate and the methanol-amphiphile stock solution was added to a final concentration of 60 mM of 2:1 DA:DOH. Fluorescence was also tracked every 30 min. For each analysis, fluorescence of vesicle samples was divided by the fluorescence of calcein buffer to account for a reduction in fluorescence over the period of incubation and transfer.

### Nile Red

Using a BioTek Synergy HT4 microplate spectrophotometer (Agilent Technologies, Santa Clara, CA, USA), we tracked emission at 610, 640, and 660 nm following excitation at 480 nm every generation. Intensity of fluorescence at 640 nm is proportional to the surface area to volume ratio of the particle stained by Nile Red.^26^ Meanwhile, the ratio of intensity at 610 to 660 nm, or the blue shift of the Nile Red emission spectrum, is an indicator of micropolarity of the system.^27^

### Tests to Assess Vesicle Disruption during Resuspension

Fluorescence-based assays using Rhodamine B and SDIP/EU^3+^ were measured with a BioTek Synergy HT4 microplate spectrophotometer on 200 μL samples containing 100 mM amphiphiles. Rhodamine B (Neta Scientific, Marlton, NJ, USA) was prepared at 6 μM concentration in a 1 M amphiphile methanol stock. Its emission was determined following excitation at 545 nm before and after resuspension. To introduce SDIP into vesicles, 1 M amphiphile methanol stock with 20 mM SDIP (Biotium, Fremont, CA, USA) was allowed to incubate for 24 h in microplates. Then, 40% of this vesicle population was transferred into PB containing 0.2 mM EuCl_3_ (Biotium, Fremont, CA, USA). Before and after resuspension of this mixture, emission of the Eu^3+^/SDIP complex was collected at 610 nm following excitation at 280 nm.

### Dynamic Light Scattering

To explore changes in vesicle size across generations, we conducted a short FR transfer experiment in cuvettes. For generation 0, 100 mM DA:DOH vesicles were prepared using 100 μL of the 1 M methanol stock and 900 μL of PB in 8 replicate cuvettes. They were measured at 0 and 24 h and again following transfer of 400 μL, with resuspension using a p1000 pipet, into new cuvettes containing 540 μL of PB. Then, 60 μL of the amphiphile stock in methanol was added to the resulting generation 1 samples, which were measured at 0 and 24 h as well. To obtain Z-average radii, dynamic light scattering (DLS) measurements were done with a Zetasizer Nano (Malvern Panalytical, Malvern, UK) with 175° backscattering at 25 °C.

## Results and Discussion

### Vesicles Respond to Selection for Greater Turbidity When Resuspending and Feeding with Amphiphile Stock

To test whether vesicle populations can respond to selection for greater turbidity, we compared changes in turbidity between selection and drift lines. In all three replicate FR experiments, a significant difference in turbidity between selected and drift lineages was observed in most generations (Figures 2A and S1A–C). For example, in the 20-generation experiment, denoted FR1 (Figure S1A), we observed an increase in the drift-selection turbidity difference from generation 3 to 10, after which the turbidities of selection lineages were generally greater than drift lineages. FR selection lineages showed consistently lower heritability than drift lineages (estimated as the regression slope of parent on offspring turbidity) (Figure 2E). These results show that heritability existing at the start of the experiment is diminished in lineages subject to selection and is not replaced. In the presence of selection, fewer of the original (generation 0) wells survive than under drift, indicating that both descendants of a given parent are more likely to survive (i.e., be selected for) in the selected lineage than in the drift lineages (Figure S2A).

**Figure 2:**
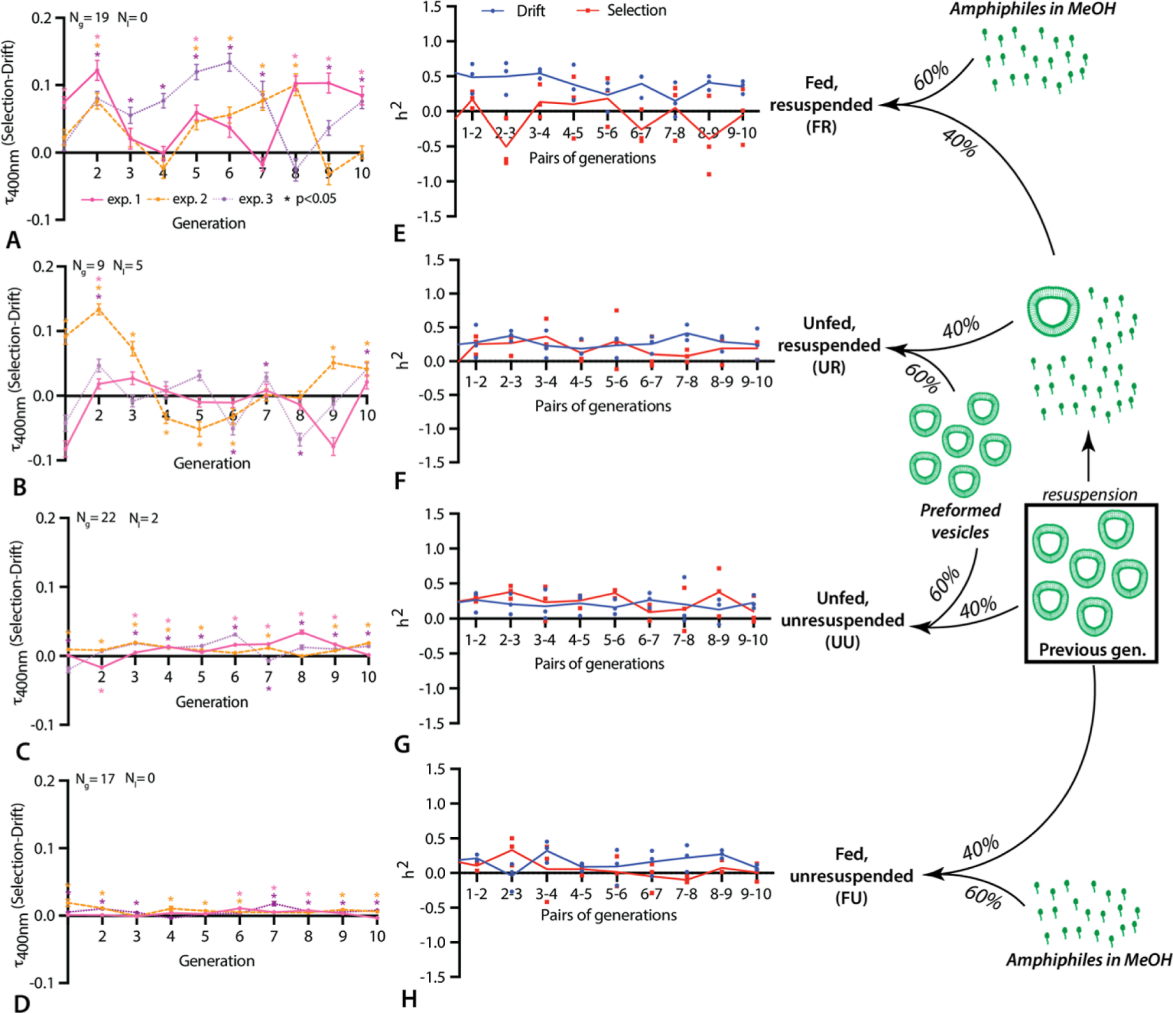
Summary of the results from the selection experiments of four different types. (A–D) are the selection-drift turbidity difference for the three replicate experiments of the FR, UR, UU and FU protocols, respectively (*n* = 96, error bars are SE). (E–H) are parent-offspring heritabilities for each pair of generations for each replicate experiment (*n* = 3, lines connect means). *N*_g_ refers to the total number of generations across the three replicate experiments where selection turbidity is significantly (*p* < 0.05) greater than drift, and *N*_l_ is the number of generations where it is significantly lower. Asterisks indicate *p* < 0.05 for the selection and drift comparison in (A–D).

To see if the response to selection for greater turbidity entails a change in population properties, such as the vesicle surface area to volume ratio or the distribution of different particle types,^26,27^ we tracked Nile Red fluorescence (both emission, *I*_640_, and the degree of blue shift, *I*_610_/*I*_660_) in one FR experiment. There was no clear pattern for either metric (Figure S3), and there was no correlation between turbidity and Nile Red fluorescence for any of the generations (Figure S4 and Table S1). This suggests that the observed response to selection is unlikely to entail changes in the concentration of amphiphiles. Although there were statistically significant differences between drift and selection lineages in some generations, the lack of temporal patterns suggests that these reflect day-to-day variation in the experiment or, perhaps, a transfer dependency of Nile Red emission, similar to that observed previously.^22^

### Removing Resuspension or Feeding with Amphiphile Stock Reduces the Response to Selection

To see if the formation of new vesicles from the amphiphile stock at the start of each generation was needed for a response to selection, we conducted triplicate UR experiments, in which amphiphiles are replenished with preformed vesicles rather than as a methanol solution. Using this UR protocol, only nine generations have significantly (*p* < 0.05) greater turbidity in selected lines (*N*_g_ = 9) (Figures 2B and S1D–F). Moreover, selected lineages have significantly *lower* turbidity in five generations (*N*_l_ = 5), suggesting a lack of a consistent effect. Additionally, the turbidity difference between the selection and drift lineages is very small and there was no difference in heritability between selection and drift lineages (Figures 2F and 3A). It is worth noting that methanol, the solvent used to introduce amphiphiles, cannot explain differences among experimental setups, since both stock-fed and vesicle-fed setups contained the same methanol concentration. These results suggest, therefore, that the response to selection in FR transfers depends on new vesicle formation (or growth and division) after the parental vesicles are fed with dissolved amphiphiles.

**Figure 3:**
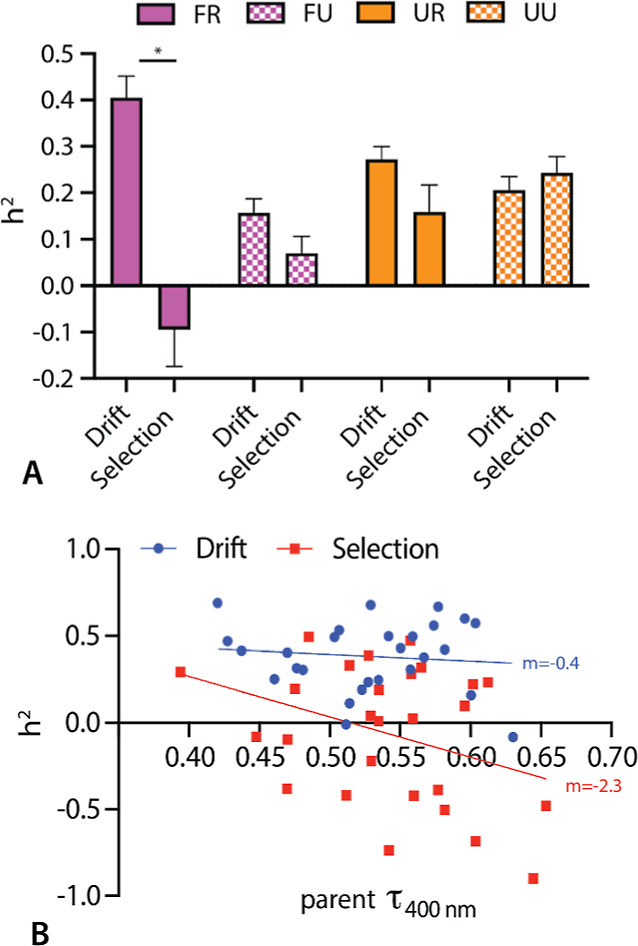
Patterns of heritability changes in different experiments. (A) mean heritability in drift and selection lineages across all generations of the four experiment types and (B) linear regression between mean parent generation turbidity and heritability of the respective parent-offspring generation pair across three replicate FR experiments. For A, *n* = 30 and error bars are SE, asterisk indicates *p* < 0.05 for Welch’s *t* test. For B, slope values are indicated on the graph.

To evaluate whether resuspension prior to transfer is necessary for the response to selection seen in FR experiments, we conducted triplicate FU experiments. In 17 of the 30 generations, turbidity is significantly greater in selected than drift lines, similar to the 19/30 significant values seen in FR (Figures 2D and S1J–L). However, the magnitude of the FU selection/drift difference is much smaller than for FR and there is less difference in heritability (Figure 2H) or in the number of surviving lineages (Figure S2D). One possible explanation for the observed significant, but minimal, response to selection is simple carryover, where offspring wells coming from more turbid parental wells are more turbid simply because 40% of their volume came from the parental well. Simple carryover is likely to be more marked in FU than FR because, without resuspension, we would expect a larger fraction of the previous generation’s vesicles to remain intact and contribute to offspring turbidity.

Finally, we conducted a set of UU experiments, where intact vesicles from the parental generation are mixed with preformed vesicles. As with FU, we observed a consistently greater turbidity in selection lineages (Figures 2C and S1G–I), but the magnitude of the turbidity difference was small and heritability did not differ significantly between selection and drift treatments (Figure 2G). These results also likely resulted from simple carryover. To evaluate whether vesicle–vesicle interactions might be contributing to the (modest) response to selection in UU experiments, we conducted a fourth experiment for five generations with each transfer done immediately after mixing, without any incubation. The lack of an incubation period in this experiment would presumably reduce interactions between the parental and newly introduced vesicles. This rapid UU procedure yielded a similar response to selection as the 24 h per generation UU procedure (Figure S5), consistent with the hypothesis that selection-drift differences seen in the UU protocol are due to simple carryover.

Overall, we observed that protocols without resuspension, where intact vesicles are transferred between generations, have a response to selection that is likely only due to simple carryover. The FR protocol had the clearest response to turbidity selection and was also the only protocol showing markedly lower heritabilities in selected lineages (Figure 2B). In the absence of selection, FR showed the highest average heritability of any treatment, whereas selected lineages had the lowest average heritability among all treatments (Figure 3A). FR also demonstrates a clear inverse relationship between heritability and mean turbidity (Figure 3B). These findings suggest the possibility that while the carryover effect is present in all protocols, or at least those that lack resuspension, the response to selection seen in the FR protocol reflects an inheritance-like process where the properties of post-resuspension vesicles are somehow transmitted to newly formed vesicles produced from amphiphiles following transfers.

### Resuspension Induces Vesicle Reorganization

We used a variety of methods to elucidate the mechanism underlying the response to selection seen in FR experiments. Fluorescent microscopy revealed that resuspension greatly reduces the number of large vesicles (Figure 4A,B), as confirmed by dynamic light scattering, which showed a significant reduction in average vesicle size (Figure 4C). These changes in the assembly size distribution likely explain the immediately increased turbidity that occurs following resuspension (Figure 4D).

**Figure 4:**
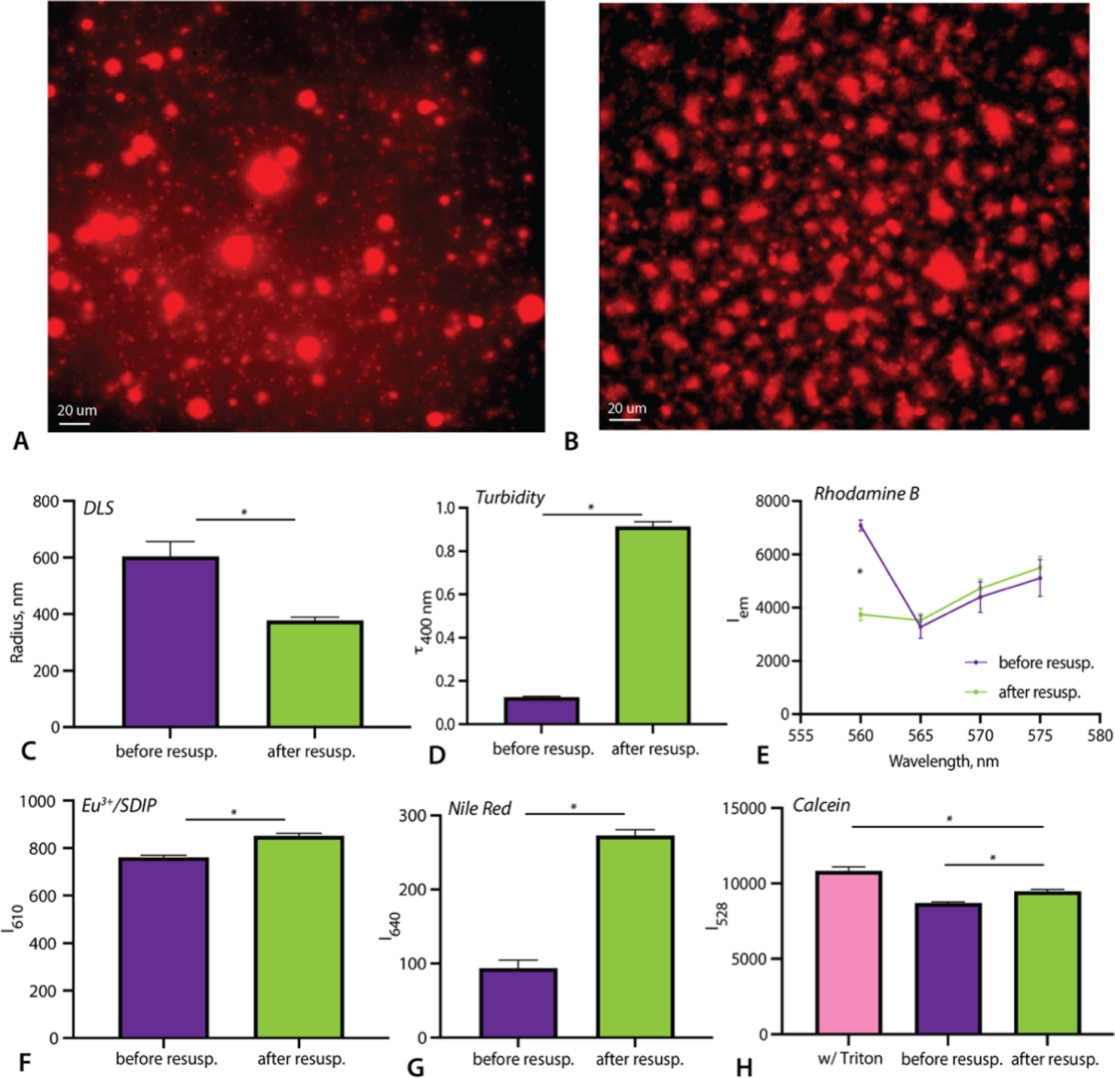
Changes occurring during vesicle resuspension. (A) Microscopy image of rhodamine-stained vesicles after 24 h of incubation (before resuspension). (B) Microscopy image of rhodamine-stained vesicles after 24 h of incubation and then resuspension: (C–H) physical changes in vesicle populations before and after resuspension: average vesicle radius (C, *n* = 8); turbidity at 400 nm (D, *n* = 96); rhodamine B fluorescence at 545 nm excitation (E, *n* = 24); SDIP/Eu^3+^ fluorescence at 610 nm (F, *n* = 3); Nile Red fluorescence at 640 nm (G, *n* = 8); and calcein fluorescence at 528 nm before and after resuspension and a positive control containing 1% Triton X-100, (H, *n* = 10). Error bars are SE. Asterisks indicate comparisons that are significant with Student’s *t-*test (*p* < 0.05).

To evaluate whether resuspension disrupts vesicles, we measured Rhodamine B fluorescence, which is known to diminish when rhodamine escapes into an aqueous environment.^28^ A test of rhodamine B fluorescence in different solvents without amphiphiles confirmed this prediction (Figure S6). Rhodamine fluorescence in vesicle solutions was lower after resuspending, which supports the hypothesis that vesicle membranes had been damaged (Figure 4E). We also used the Eu^3+^/SDIP chelating assay, developed by Biotium (Fremont, CA, USA), with SDIP added to the input population and europium provided in the dilution buffer added after transfer. This revealed an increase in fluorescence following resuspension (Figure 4F), indicating Eu^3+^/SDIP complex formation and further showing vesicle disruption. Other evidence of vesicle disruption during resuspension comes from higher Nile Red emission at 640 nm, which suggests that resuspending causes an increase in the vesicle area-to-volume ratio (Figure 4G).

We also used DLS to track vesicle size over the course of incubations in generation 0 and 1 samples. In generation 0, over the course of a 24 h incubation (without any prior generations), vesicle radius increases slightly but not significantly (*p* = 0.13; Figure S9A). During generation 1, vesicle size in FR samples increased noticeably to 900 nm (Figure S9, B). It should be noted, however, these experiments were done in 1 mL volume in cuvettes, so these results may not be comparable to the FR experiments in 200 μL 96-well plates.

We observed that calcein fluorescence in prepared vesicle solutions declined immediately after resuspension and continued to decline over the next 3 h (Figures 4,H and S7,S8). This likely indicates leakage and/or breaking of the vesicles induced by resuspension. It should be noted, however, that removing unencapsulated calcein in this analysis is impossible because size-exclusion chromatography affects vesicles of this composition and increases turbidity in a manner similar to resuspension. However, since the presence of the vesicle-disrupting detergent Triton X-100 causes an increase in calcein fluorescence (Figure 4H), we can conclude that there is still a fraction of intact vesicles left after resuspension. A similar pattern to these generation 0 results is seen in the time series of generation 1, following FR transfer, although the equilibrium state is achieved in a slightly longer time (Figure S8).

### A Response to Selection Depends on a Period of Interaction between Parental Material and Dissolved Amphiphiles

Except for the UU protocol, turbidity was dynamic over the course of the first incubation, showing a transient increase and then a steady decline (Figure S10). The FR and FU protocols showed a marked peak of turbidity within the first hour, which was absent when the amphiphile stock solution was not added (Figure S11). In the FR protocol, which combines resuspension and methanol stock feeding, there was a second increase in turbidity between 2 and 6 h that is absent from the other protocols. This led us to suspect that in this time period, there may be a cooperative process, where an interaction between transferred vesicles and introduced food influences the resulting vesicle population.

To test this hypothesis, we conducted multiple FR selection experiments with varying generation times (Figure 5). With transfers every 0.5 to 1.5 h, there was no clear increase in turbidity in selected lineages relative to drift lineages, and, moreover, overall turbidity decreased after the first 1–3 generations (Figure 5A,B). This indicates that, at those time points, the amphiphile stock does not yield enough new structures to prevent serial dilution of the initial turbidity (the initial generation was preceded by a 24 h incubation). With 5 h of incubation, there was no turbidity decrease and 3 out 5 generations showed elevated turbidity in selected populations (Figure 5C), similar to that seen in the 24 h transfer (Figure 5D). This suggests that a process enabling heritability of turbidity, thus contributing to the response to selection, begins between 1.5 and 5 h. This time window corresponds to an almost linear increase in turbidity (Figure 5E), which is absent in time courses without amphiphile stock addition (Figure S11). Since turbidity scales with the logarithm of vesicle size, this linear increase is probably indicative of increases in the vesicle concentration rather than vesicle size.^24^

**Figure 5:**
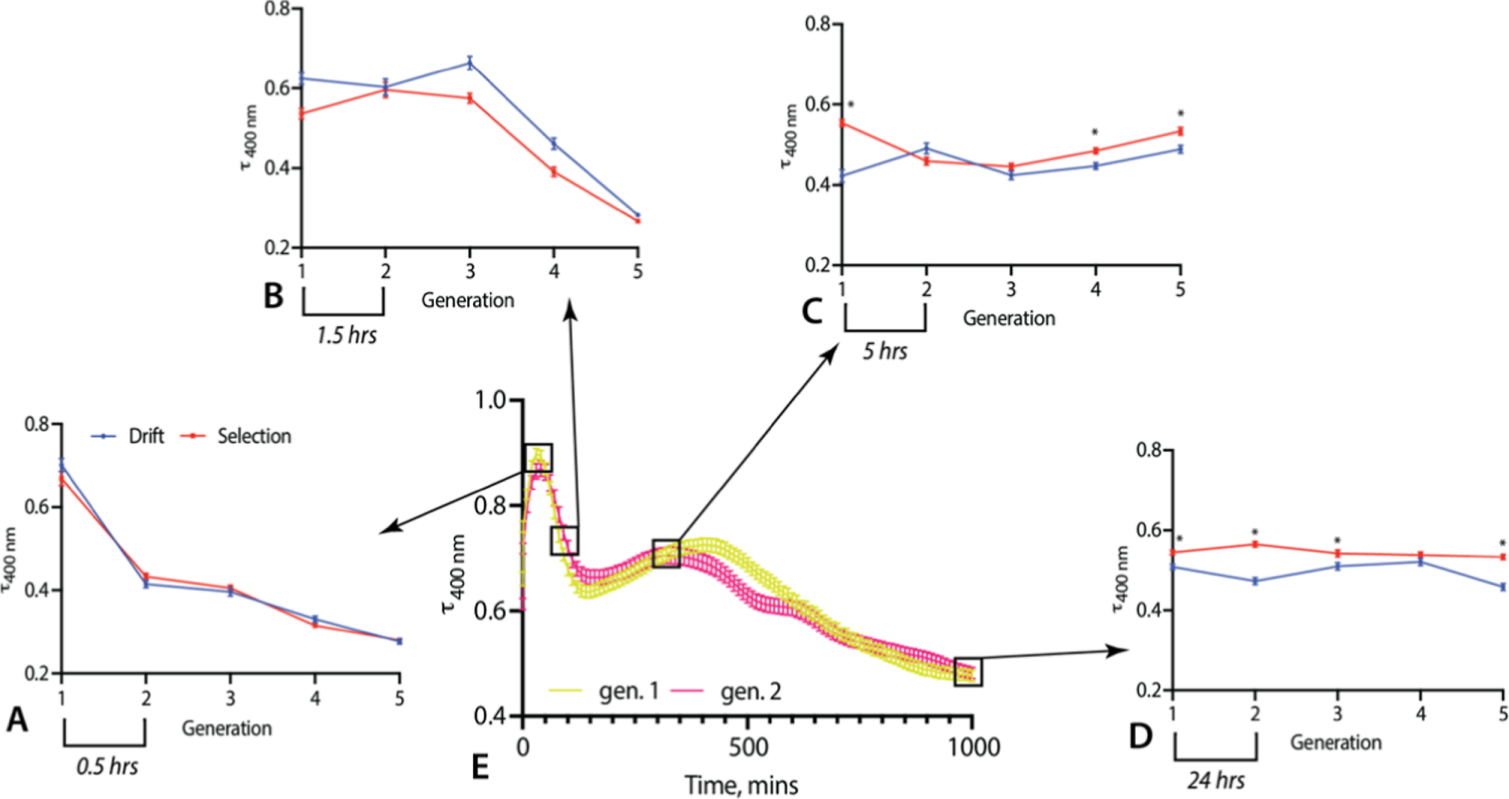
FR selection experiments with varying length of incubations: (A) 0.5 h, (B) 1.5 h, (C) 5 h, and (D) 24 h (the latter is derived from the average of the three replicate experiments shown in in Figure 2A). (E) Time course of turbidity changes in the first two generations of an FR transfer, with time points used for the 5-generation transfer experiments boxed. Asterisks indicate *p* < 0.05 between selection and drift. *n* = 96, error bars are SE.

### Emergent Vesicle Dynamics Supports a Nontrivial Response to Selection but Does Not Enable Open-Ended Evolution

Considering the persistence of some transfer vesicles through resuspension and the high heritability seen at the start of the FR experiments, there are 4 plausible processes that may be occurring following introduction of the amphiphile stock: (1) cooperative vesicle assembly where de novo vesicle formation from food molecules is sensitive to characteristics (such as size) of the preexisting vesicles; (2) independent assembly of food into vesicles followed by spontaneous fusion/fission interactions with the transfer vesicles; (3) growth of transferred and newly formed vesicles via monomer or micelle incorporation; and (4) breakdown of transfer vesicles and assembly of new vesicles from pooled food amphiphiles. Based on the lack of response to selection seen with 1.5 h incubations but positive response seen with 5 h incubations, heritability must be based on processes that begin after 1.5 h of incubation. The linearity of the turbidity increase between 1.5 and 6 h suggests that de novo vesicle formation is the most likely process, however, this must be verified with additional studies. Prior to this time point, the rapid increase and then decrease of turbidity likely correspond to the formation and dissolution, respectively, of nonvesicle aggregates. This could be an explanation for the decrease in turbidity seen with 30 min incubations (Figure 5A).

Due to the large volumes required for DLS, we were unable to directly track vesicle size over the course of each experiment. However, Nile Red peak emission data (Figure S3A) suggest that it is unlikely that, after generation 0, vesicle size changes significantly over the course of the FR experiments.

In FR, FU, and UU transfer protocols, we see consistently higher turbidity in selection lineages compared to drift controls. The increase in turbidity seen in FR experiments resembles responses to artificial selection seen in biology insofar as it is associated with a loss of heritability.^29^ The failure of heritability to decline in FU and UU experiments suggests that the response to selection in these cases may have a different mechanism than FR.

In the FU protocol, many generations show increased selection lineage turbidity (17 compared to 19 in FR; Figure 2D), but the absolute difference between selection and drift heritability, as well as the mean drift heritability are the lowest of all experiments (Figure 3A). This protocol allows a large population of vesicles from the parent to influence the formation of new vesicles forming from dissolved amphiphiles. One might expect, therefore, a stronger response to selection than was seen with the FR protocol. However, an inverse relationship between template vesicle concentration and heritability of vesicle size was observed previously for the matrix effect.^17^ This aligns with a proposed mechanism for the effect, which requires overloading of the outer leaflet of seed vesicles when there is a large excess of unincorporated amphiphiles. This is proposed to lead to a doubling in vesicle radius followed by division into two equal vesicles, resulting in the “inheritance” of vesicle size.^17^ This would explain the stronger response to selection seen in FR than FU experiments, which is consistent with the matrix effect, or a similar mechanism, occurring with the FR protocol.

In UR and UU transfers, 60% of the solutions contained vesicles that were prepared independently every generation. This limits the potential for a parental sample to influence the next generation in any way other than through simple carryover. These experiments, when compared to their respective stock-fed experiments, thus provide a baseline selection drift and turbidity difference. In UR experiments, there is no consistent response to selection: 9 generations have significantly greater turbidity in selected lines (*N*_g_ = 9), whereas 5 have significantly lower turbidity (*N*_i_ = 5) (Figure 2B), contrasting greatly with *N*_g_ = 19 and *N*_l_ = 0 for FR experiments. This suggests that carryover effects with resuspension are minimal and cannot account for the observed response to selection in the FR protocol. Similarly, we conclude that the selection/drift difference in experiments UU1-3 (Figure 2C) is most likely explained by carryover. Although it is conceivable that fusion-fission dynamics occurring when two populations of vesicles are mixed could induce some form of inheritance, this does not seem to be a factor here, since we observe a similar response to selection when the generation time is reduced to 30 min (Figure S5). This would make vesicle–vesicle interactions extremely unlikely, especially since turbidity does not change during incubation in the UU setup (Figure S10). Taken together, these data suggest that observed selection-drift turbidity differences in unresuspended experiments are the result of simple carryover rather than being due to true inheritance of vesicle properties from parental to offspring generations.

Comparing the results from all experiments, we can confidently claim that while the response to selection seen in UU and FU is largely a trivial outcome of vesicle carryover, the response seen in the FR is due to inheritance-like dynamics, likely involving vesicle growth and division and/or new vesicle formation. This heritability is apparently stronger after vesicle populations are resuspended, which results in a lower fraction of intact vesicles being transferred, consistent with a matrix effect-like mechanism.^17^

## Conclusions

These results are the first definitive evidence that lipid vesicles can respond to selection for their physical properties in a manner similar to that of extant biology but without genetic molecules. However, the response to selection seen was immediate and short-lived. There is no evidence, even after 20 generations, that new heritable variation emerges, suggesting a lack of the open-endedness that characterizes biological evolution. Nonetheless, it is striking that the FR experiments demonstrate nontrivial heritability, where information passes from parental to offspring generations by means other than simple carryover.

To better understand the mechanistic basis for the response to selection in FR experiments, future work could involve molecular dynamics simulations. Experimentally, it would be desirable to make real-time observations of vesicles that have been resuspended when they are fed with new amphiphiles. Additionally, it would be interesting to target other amphiphile species or utilize different feeding strategies, for example, supplying micelles instead of amphiphiles dissolved in methanol, to determine whether nontrivial inheritance is sensitive to these factors.

The mechanism for the observed response to selection appears to involve physical interactions among vesicles rather than the compositional inheritance assumed by the GARD model. As our experiments only include 2 amphiphile species (excluding ionic states), the possibilities of compositional inheritance seem limited. However, our experimental framework could be readily deployed in cases where there is a much greater diversity of amphiphiles present to determine if a more open-ended response to selection is seen. Such a result would go a long way to supporting the hypothesis that compositional inheritance played an important role in prebiotic evolution prior to the acquisition of nucleic acid polymers.

## Supplementary Material



## Data Availability

All data obtained in this study and used to create figures is deposited in DataDryad at the following link: https://doi.org/10.5061/dryad.fbg79cp99

## Notes

Figures and tables with additional data from the experiments described in this study : selection and drift turbidites for each experiment; number of generation 0 lineages with surviving descendants in each generation; results of Nile Red and calcein fluorescence measurements; raw turbidity data from short transfer experiments and time courses; and changes in vesicle size during incubation (PDF)
